# Elements of General Pathology

**Published:** 1845-10

**Authors:** 


### ART. IV.

*Lehrbuch der Allgemeinen Krankheitslehre.* Von Dr. CARL H. SCHULTZ, &c.—*Berlin,* 1844-5.

*Elements of General Pathology.* By Dr. C. H. SCHULTZ, Professor in the University of Berlin, &c. &c.—First Part, 1844. Second Part, 1845.—*Berlin,* 1844-5. 8vo, pp. 738.

NOT long ago we took an opportunity when we were quite in the humour for the work, to examine analytically the doctrines of Professor Schultz, as to the moults and renewals of the blood and tissues. It is in our Sixteenth Volume that we have erected this memorial of our patient endurance of toil. In the present article, we propose to raise a monument of our perseverance by presenting to our readers the principal of those points in pathology to which the Schultzian doctrines are applied by their author.

We premise introductorily, that Prof. Schultz aspires to be a radical reformer in medical doctrines and practice. He proposes (to use his own words) to transfuse the old inorganic spirit of medicine into the organic, and unfold to the unfettered understanding the wonders in the doctrines of life and death and healing. In prosecution of this purpose he heartily attacks existing doctrines, while he makes room for his own. The first onset is in a preliminary dissertation on the present condition of medicine, and on its relations to the sick and to the commonwealth. On looking at the future from the present, the tree of medicine is seen towering like a sciential Colossus, and dwarfing beneath its shade the first shoots of the future. So Professor Schultz thinks; and also that there can be no hope for the development of the future unless light stream upon it through the colossus that ages have reared. He therefore hews at its massy trunk, and drives his wedges into the deep chinks that time has laid open; he barks its gnarled branches; he tears away the old but green ivy that wraps its rottenness to the topmost boughs. He does all this freely too: for (as he observes) we live in civilized societies and in a time when scientific not less than political freedom is as necessary a condition of life as the pure air of heaven.

But if Professor Schultz insists that scientific principles should guide practice, he does not despise the lessons of experience. He utterly abhors the self-styled practical men, who to cover their ignorance, glory in their empiricism. Experience might be blindly followed with safety if it were true, that the same cases recurred again and again, but a thousand variations are seen in the progress from life to death, the causes and origins of which have yet to be investigated. Experience herself teaches us that experience is not sufficient. Man therefore seeks continually after the surer light of general principles, and the sick will suffer in proportion as these are false. Principles must be tested by experience; but it is better that the mind be kept excited by antagonising propositions than be stupefied by gross empiricism.

Principles of the most opposite tendencies have, however, accumulated in medicine; the chemical, dynamical, physical, mechanical are all in active existence. Errors and truths are mingled. The crab creeps out of his old shell, and the snake escapes from its sordid skin, but medicine is powerless to disburden itself of its effete doctrines. It wants complete renewal, an efficient moulting. This process Professor Schultz is determined to effect.

The First division of the work is occupied with a discussion and refutation of the erroneous doctrines referred to. The chemical theory of respiration and Liebig's views are duly criticised as unsatisfactory and erroneous, both theoretically and practically. Nor does rational physiology receive greater mercy at his hands. In vegetative life, it considers the phenomena as chemical; in animal life as dynamical, as if it were possible, he observes, that two such opposite principles could rule together. These and similar notions of our author we need not discuss. It has been weary work to us who never trouble our readers nor ourselves with discussions of the kind, to read the book. It may be true, as he asserts, existing physicians wear the cast-off wigs of Hippocrates and Diocles Carystus as if they were of the newest fashion; it is our duty to examine the structure and *mode* of Professor Schultz's wig.

*The new researches.* Under this title Professor Schultz comprehends his own researches. To these new inquiries belong, our author states, that on the vegetation of disease, and on the natural consanguinity of disease and vegetable life. This inquiry has a decided bearing on the more intimate knowledge of the death-process in disease. . . . . . Plants have an external renewal only (anaphytosis), the inner being wanting. To this state the sick body of man retrogrades, since in it the normal renewal is so interrupted that the two processes of formation and moulting (the formation and excrementitial plastic), as in plants, are intermingled. But the sick body cannot counterfeit plants in other respects by anaphytosis, and it is through this inner restraint on the renewal-process, that the death-process is set up. We prefer giving the somewhat uncouth words of our author as the clearest, although literal expression of his meaning. Disease has a personality given to it. It makes numerous efforts to break out into vegetative life, as in exanthemata, &c. Many botanical terms have, indeed, been introduced into pathology, as fungus, taking root, efflorescence (blooming), eruptions. This is the first of the curls, in his new wig, which Professor Schultz invites his medical friends to inspect and appreciate. And yet, although we decline travelling through the whole round of seventeen new inquiries instituted by the author, we find something worth our gleaning. We will first notice some of his etiological views.

*The inherent predisposing causes of disease.* (Anlage.) These are organical conditions of the system which operate not in harmony with external causes, but in opposition to them. They are not the cause but the basis or substratum of disease. The true and only cause of disease is the death-process and death-power, as the vital power and vital process constitute the cause of life. These causes are to be strictly distinguished from the *conditions* of disease. The predispositions (anlage) under consideration consist in an incongruity in different organic systems and functions of the body, particularly in a change in the normal harmony of acts, as in the healthy oscillation of renewal and moult. There are two general divisions comprising numerous subdivisions. Firstly, there is an abnormal preponderance of single organs and functions over others. In the vascular system, the heart may preponderate over the capillary system, or the spinal cord over the brain in the nervous system, or the lungs may be incongruous with the vascular system. Secondly, there may be an incongruity between the two renewal-processes of formation and moulting; this may occur in any system and hinder the free course of vital acts within it.

The following are some of the details of the predispositions to disease laid down by Professor Schultz.

*Predisposition in the intestinal mucous membrane.* This mucous membrane casts off its epithelium or moults like the skin, the only difference being that the moulted matter from the latter is dry, while from the former it is mixed with water, and forms mucus. If the excreted mucus thus moulted accumulate, it constitutes a local irritant, giving rise to thickening and inflammatory irritation, and forms a nidus for the reception and development of intestinal worms. Where the excreted matter is retained from debility of the muscular fibre, it acts also as an irritant upon the spinal cord, inducing excito-motory phenomena. It may also interrupt the functions of the membrane, and so a healthy assimilation be prevented, and a morbid state of the blood induced.

*Acidity.* Our readers will remember that on a previous occasion, (vol. XVI, p. 221,) we detailed Prof. Schultz's doctrines as to the acids found in the stomach, especially in the gastric juice. These doctrines are here re-asserted. The gastric juice is not secreted by the stomach, but is a mixture of sour chyle, saliva, and mucus. In a healthy condition of the digestive organs little acid is formed, but when they are weak, a chemical decomposition of the food takes place, and free acid is formed. This decomposition is partly consequent on diminished vital power in the salivary glands and intestinal canal, partly on a defective and vitiated biliary secretion. In consequence of this circumstance, the chyle in the duodenum is not sufficiently de-acidified and vital action cannot preponderate over chemical. Acidity is not confined therefore to the stomach, but may extend through the whole canal, predisposing to diarrhea and gastro-enteritis, as well as to softening of the stomach.

*Predispositions in the lymphatic system.* We pass over some minor points in the pathology of the alimentary canal to notice this head. The whole vascular system is to be considered as a very fine alimentary canal, whose anterior opening corresponds to the lymphatic, and the posterior to the portal system. Consequently, the former is in close relation with the blood; indeed lymph must be considered to be blood in an inferior stage of development, as in the articulata, mollusca, &c. The lymphatics take up fat and albumen from the intestinal canal; the former is the element from which globules are formed, the latter the basis of the plasma. Fat is the product of chylification, and of special kinds of food. The morbid predisposition of the lymphatics can consist only in an irregular formation of albumen or fat. If the former predominate, the lymph is transparent, pale, readily coagulable; but it is milky if fat predominates, and coagulates slowly. As the lymphatic glands act like plasma upon the lymph and initiate the formation of the blood-vesicles, if the functions of the glands be impaired, the latter are imperfect.

*Predispositions in the blood.* Renewal (ananeosis, from the Greek word

*ἀνανέωσις*, renovatio,) and moulting are the two principal points with which pathology is connected. Ananeosis or renewal of the blood begins in the lymphatics, as we have already seen. In the lymphatic glands are formed the vesicles which surround the globules, and which are subsequently transformed into blood-vesicles. These vesicles possess irritability and contractility, and in virtue of these properties absorb oxygen, form red colouring matter, work up their nuclei, and develop the plasma. If the vesicles be imperfect, all these changes are imperfectly performed. Their respiring power being impaired, the plasma is imperfectly formed, the colouring matter deficient. The vesicles are pale, chlorotic. When the blood-vesicles are imperfectly contractile, the colouring matter is imperfectly retained. It thus passes out into the plasma, and reddens it, constituting that state which is the normal condition in red worms, molluscs, &c. The plasma is thus rendered capable of absorbing oxygen, and so it excites the nutritive process in organs too strongly. Hence blennorrhœal and glandular inflammation. In chlorotic subjects the vesicles are flabby and incontractile. Ananeotic blood may have vesicles too irritable and contractile. In this state they absorb an excess of oxygen, and stimulate the organs. This condition occurs particularly about puberty contemporaneously with the more rapid development of the lungs. Professor Schultz calls it the neoteretic state (neotish-eretisch.) All the successive changes in the blood-vesicles take place rapidly. They never become old, but are emptied early and moult unripely, constituting an antagonism to the melanotic condition. This neoteretic condition is the basis of the sanguineous temperament: it is also very strikingly perceptible in phthisis, and explains, according to Professor Schultz's ideas, the cerebral activity and sensual quickness observed in the phthisical.

The formation of the plasma may be imperfect; it may contain too much albumen, and consequently too little fibrine. Thereby the blood moves less rapidly, is less stimulant to the vessels, and so passive congestions, and vascular dilatations, particularly of the veins, take place. But on the contrary, the plasma may contain an excess of fibrine and then a diametrically opposite class of pathological phenomena are seen. Nutrition is excessive, and therefore, hypertrophy, active congestions, and inflammations appear. The process of blood-moulting is the basis of several pathological states. We refer our readers to our previous account of the moulting process. In moulting, the vesicles may appear in various states. They contain a large quantity of dark colouring matter, and so their specific gravity is increased, and they easily sink; the membranes are flabby and incontractile, allowing the colouring matter to escape through them: they are unirritable, and are not therefore acted upon by the air and made redder, and finally, their nuclei are quite dissolved away. The blood may be dark in two ways; in the embryo, for example, it is dark from the want of oxygen, and not from the presence of useless vesicles. The arterial blood in the placenta is to the fœtus what water is to the fish; the oxygen is supplied scantily by this mode, but so soon as the full pulmonary respiration takes place, the blood changes to a brighter red. Blood may be dark because the supply of oxygen is cut off: this is venous blood and constitutes the "venous state" of Puchelt; or the blood may be dark from imperfect moulting and depuration: this is melanotic blood.

Now the liver is the porta or gate through which the blood is expurgated from the black perilous stuff dissolved in the plasma of melanotic blood. It passes off as bile. But the plasma itself is smaller in quantity, and scarcely stimulant to the blood-vessels, and so the blood moves slowly through the portal system. It moves slowly at all times, but more slowly when the liver is inactive, and the perilous stuff is partially retained. Thus congestion of the portal system, or in other words "abdominal infarction," occurs: and a reflux taking place to its minutest roots, the hemorrhoidal vessels become dilated, and so hemorrhoids arise. The spleen participates in the slow motion of the blood through the portal system, but its office being that of a lymphatic gland with reference to the fluids taken into the system, its morbid changes are analogous to those observed in the other glands of the intestinal canal.

A stoppage of the depurative process in the liver may originate changes in remote parts also. The whole mass of the blood gets charged with the perilous stuff; and 1. The dead vesicles show a tendency even to chemical decomposition, as in cachectic hemorrhages, melæna, stinking secretions, nauseous cutaneous affections. 2. The melanotic blood acts injuriously on the muscular and nervous systems; it is deficient in the stimulating property of healthy blood. Thus the brain, sensual nerves, and muscles, are imperfectly acted on, are weakened, and at last paralysed. Apoplexy, intermittent fever, spectral illusions, and even paralysis of the senses are the result of this state. Melanotic blood will also act on the organs of vegetative life. It is this property that distinguishes it from venous blood. The former always develops cachexies, the latter never.

*Predispositions in the vascular system.* The vascular system is particularly characterized by irritability (we write as our author writes,) but this is shown in varying modes in different portions. It is exhibited in the highest degree in the venous system. The veins are so strongly contractile that they will contract even to the closure of their cavity. Hearts form at almost any point in the veins, as is exhibited in these animals which have several hearts. Arteries differ from veins in this, that their contractility is simply that of cellular tissue; they have great elasticity, but do not contract strongly; arteries have less vital irritability. These characteristics are the foundation of the varying forms of disease presented by the two classes of vessels upon which Professor Schultz duly speculates. We leave these and the like unnoticed to discourse on the

*Predispositions in the liver.* The biliary secretion is influenced by that of the intestinal canal. The actions of the gall-bladder and biliary ducts sympathize with the movements of the latter. In diarrhea bile is excreted more rapidly, in constipation more slowly. Infarction of the liver predisposes to the formation of gall-stones and to hypertrophy.

*Theory of tropical livers.* Hepatitis, yellow fever, bilious vomiting and bilious diarrhea are common in summer and in tropical climates. The predisposition to these consists in the transformation of melanotic into bilious blood primarily in the liver. To understand the origin of this, it is necessary to remember that there are two principal requirements for the due secretion of bile, namely, 1. Colouring matter in the used-up blood-vesicles concurrently with masses of black cruor incapable of acting on the atmosphere or of vital action. 2. That the colouring matter shall be no longer fixed in the blood-vesicles, but dissolved in the plasma. On its passage through the liver, the portal blood in the healthy condition loses the colouring matter contained in the plasma, it being used in forming the bile.

The cause of this readier solution of the colouring matter in the plasma of the portal blood is to be found, partly in the flaccidity of the membrane of the vesicles, partly in the greater wateriness of the portal blood. Now these two conditions of the blood (the causes of bilious blood) originate in the mode of diet during the heats of summer, or in tropical countries. Firstly, the diet is principally vegetable; secondly, water is drunk to a great excess. That a vegetable diet influences the contractility of the membrane of the blood-vesicles is manifest, Professor Schultz argues from various experiments. He finds that the membrane of the vesicles is smaller and more delicate in herbivorous than in carnivorous animals. The vesicles of the latter yield up their colouring matter much less readily than those of the former. From three to four parts of water washes it all from the blood of sheep or rabbits, while from five to six parts and even more is necessary to render the blood of cats or dogs colourless. The conclusions are obvious. The blood of vegetable feeders and water drinkers most predispose to bilious disorders. The theory fits tolerably well to the problem, for which it is drawn up; and it may be true too that sheep (great grass-eaters) die often of liver complaints. But is the general conclusion true? Do other herbivorous animals so suffer, and are water-swilling, pudding-eating men liable to yellow fever, bilious diarrhea, &c.?

*Predispositions in the lungs.* Firstly, the mucous membrane of the lungs may moult imperfectly, or too quickly. In the former case, the mucus and moulted matter accumulate, and prevent the access of the air to the blood; the latter thus assumes a venous character, and is less irritant; a circumstance of some value to irritable persons. If the membrane moults too rapidly, it is then exposed naked to the air, and thus irritability is excited. This unripe moulting is usual after pulmonary affections.

Secondly, the blood and the condition of the vascular network on the surface of the lungs may constitute predispositions to disease. When vesicles enter the blood from the lymphatic system half developed, (they absorb oxygen quickly?) it is rendered stimulant, and disposes the lungs, in circulating through them, to inflammatory diseases. The blood in the embryo and infant has a similar quality. The vesicles in melanotic blood are flaccid and dead, and are little affected by the air, absorbing less oxygen in proportion as the membrane is flaccid. In this state they do not excite the capillaries to propel them on, and so they heap up in the lungs. This capillary congestion in the lungs reacts on the whole vascular system and even influences assimilation. The breathing is next oppressed, the excito-motory stimulus is not duly developed, and asthma comes on; next palpitation, venous aneurism, paralytic affections of the brain and nerves, and a disposition to bilious and melanotic excretion, even in the lungs themselves. Predispositions may exist in the machinery of respiration. Coughing, sneezing, laughing, and particularly long-continued speaking or singing, may induce congestion, &c.

*Predispositions in the glandular or secreting system.* The glands are diverticula of the mucous membrane. Their surface then must moult just as the skin, the intestinal canal, or the pulmonary mucous membrane. If this process be abnormal, secretion is at once deranged, and hypertrophy dilatations, indurations, softening, &c., occur. If the moulting process be too active, profluvia are induced. The state of the blood circulating through the secreting organs influences their diseases. Blood containing an excess of carbonic acid only, interferes little with their functions: and the morbid action of bilious blood is slow. Melanotic blood induces congestion in the capillaries, in the mode already stated when referring to the vascular network in the lungs, and hemorrhage takes place. The various changes in the plasma have a much greater influence on the secretions than the blood-vesicles. But as in coagulation of the blood the serum separates, so also it passes off through the secreting organs. In the depurative secretion, it is simply poured out; in the plastic secretions it appears as a new formation from the plasma.

*Predispositions in the nervous system.* The nerves act on the secretions by acting on the blood-vessels to which they are distributed. Of all the secretions, it is the urinary which has the closest relation with the nervous system. Spinal irritation and spasmodic affections generally have important relations also to the state of the blood. Of course melanotic or bilious blood is the most effectual in developing centric phenomena. A predominance in development of the spinal cord over the brain seems to predispose to involuntary excito-motory and spasmodic affections, as in brutes, and in Cretins, Negroes, and Mongols.

*Predispositions in the muscular system.* These appear in the motor nerves on the one hand and in the muscular fibre on the other. The condition of the latter is very much dependent on the condition of the cellular membrane, of which it is a higher grade of development. This is especially seen in the muscles of vegetative life. The skin is the organ through which the moulted debris of the muscles are excreted. Inactivity retards the renewal and moulting process in muscular fibre. The muscles themselves partly retrograde in development to cellular tissue. The blood congests in the peripheral vessels, and the circulation through the heart is less active. In consequence the respiratory and digestive functions are both impaired, and the reaction extends even to the lymphatics, and to the intestinal canal.

We shall not trace our author's views through the successive subdivisions (of age, sex, temperament, &c.) of his subject, as they appear altogether inferential, being in fact deduced from his peculiar theory.

The second portion of this (the first) part, is devoted to pathogeny, comprising an analysis of the death-process. There are three stages in the course of disease: 1, the stage of sickening; 2, of cure; 3, of death. The natural laws of disease are the laws of the contest between life and death. The first is, that the disease is death taking up his residence in the living body. Once there he endeavours to disconnect organic form and irritability, and introduce chemical matter and chemical laws. This is the morbid process. The means to obtain this end is the confusion of the formative with the secretive plastic, whereby the disease became vegetative. In the sick body, the remaining healthy life is a combat and endeavour to repel the chemism and chemical decomposition. Life guards against putridity; and so our author goes through many a weary page of theory. The few interesting points scattered through the remaining half of the volume have no directly practical interest, and we think our pages will be better occupied with them when others have confirmed the facts on which Professor Schultz builds his reform of medical science.

II. The Second part opens with a catalogue laudatory of the author's new views on the nature of fever, the pathology of the blood, the still-disputed questions of sthenia and asthenia; his theory of the pulse, and twenty similar questions, through all which we have made a critical pilgrimage, not without wonder that he should toil so and be unconscious the while that he was labouring in vain. In this second part we notice first:

*An analysis of the phenomena of disease.* The krankheitsactionen, the actions of disease, as our author chooses to term the phenomena or symptoms of a morbid state, are divided by him into—

1. Injurious or biolytic actions (actiones biolyticæ). These belong to the biolytic (life-unloosing) processes and periods of disease, and consist especially in the phenomena of interrupted or abolished function. They may be, *a,* idiopathic, as the dry skin in rheumatism, inflammation, exanthems, the pain in gout, the stupor in cerebral congestion; or *b,* sympathetic, as the impaired digestion in inflammations, the wasting in paralysis. Frequently they are antagonistic, as the dry skin in diarrhea.

2. Conservative or agonistic actions (actiones agonisticæ.) These appertain to the conservative processes and periods of disease, and consist principally in the phenomena of reaction of the central organs against the injury to the peripheral organs. The heart, brain, and spinal cord are their seat. Fever and reflex movements belong to this class. They are subdivided thus: *a.* Agonistic actions derived from the central nervous system, as cough in affections of the glottis, vomiting from gastric irritation, the straining in tenesmus, &c. *b.* Fever seated in the central vascular system. *c.* Local agonistic reaction, as in inflammation after wounds, increased secretion from irritation, as of the tears from irritation of the conjunctiva, and the like.

3. Anabiotic or restorative actions (actiones anabioticæ.) These belong to the renewing processes after disease, and consist in the phenomena of morbid new formations and morbid moults. There are two classes: *a.* Actiones epigeneticæ, or actions of new formation, occurring when the actiones agonisticæ and biolyticæ cease and the healthy processes succeed to them. *b.* Moult actions, or actiones apolyticæ, indicated by the complete desquamation of the skin and moult of the mucous membrane in exanthemata; the death of the cellular tissue (suppuration) in inflammations; the excretion of melanotic blood-vesicles after intermittents, &c.

In these "actions" Professor Schultz finds a basis for his special nosological arrangement, and for the theories adapted to each *form* of morbid action. Fever is discussed as a morbid action (actio agonistica) of the vascular system.

*Theory of fever.* Fever is defined to be the "action" by and through which local diseases become general; it is the reaction of the vascular system against disease. Many diseases may exist locally without exciting fever, but it is because they are not of a fatal character. Before they can destroy life the blood must be implicated in the morbid action, and fever set up. The true characteristic of fever is increased frequency of the pulse, and respiration. Rigors, heat, and sweats do not necessarily accompany fever. The blood is impelled from the centre to the congested periphery, and the pulse is hard in proportion to the amount of congestive resistance. The more the formative and moulting processes are interrupted, the greater the fever. On the contrary, so soon as these processes are being restored, as when the dry skin becomes moist, and the scanty urinary secretion copious, the pulse becomes slower, the heat less, the breathing more free.

*State of the blood in fever.* The febrile reaction of the central vascular system depends on an abnormal self-excitation of the organized constituents of the blood. The normal self-excitation of the vascular system depends upon the renewal of the blood within it—upon its new formation in the lymphatics, its vitalization in the lungs, its moult in the portal system; and further, on its renewal by the nutritive and formative processes going on throughout the whole body. In fever these changes are all interrupted. The vital acts of the blood-vessels go on normally no longer, and in consequence the plasma accumulates in excess, and the blood becomes anaplasmatic. A secondary result of this is, that the red colouring matter of the blood-vesicles is dissolved into the plasma constituting the red serum of fever-blood, and giving the high colour to the urinary secretion. In addition to this the moult of the tissues, as the nervous and muscular systems, &c. accumulate in the vascular system, the functions of the organs through which they are ordinarily secreted being interrupted. The heat of fever depends on the reaction on each other of the organic constituents of the blood, either locally in an inflamed part, or generally throughout the vascular system.

Professor Schultz considers the cold, hot, and sweating stages of an intermittent as excito-motory phenomena; that, in fact, an ague is a nervous fever of the spinal cord, and that pathologists err in taking its phenomena as the type of those of fever in general. The moult of the spinal cord is interrupted in intermittent fever—of the brain in nervous fever; and the one febrile affection will pass into the other by imperceptible degrees. The rigors, yawning, stretching, and other phenomena depend on the reflex action of the spinal cord while in a state of interrupted moult.

We shall not follow our author through his dissertation on the morbid actions of the vascular system. We may state shortly, however, that under this head he notices successively the different kinds of fever, palpitation, syncope, asphyxia, the morbid pulse and morbid states of the blood. Under the latter head several new and ingenious views are set forth—in direct opposition, we need scarcely remark, to the chemical theories of the day. "The enormous mass of analyses of the blood," he observes, "lie before us like the bones of a slaughtered host, heaped up high as the heavens, so that the life of which they are the offcasts can scarcely be seen." There is detailed, however, no one experiment of his own. Fibrine, according to his views, is not a chemical constituent of the blood, but a tissue first formed out of the plasma during coagulation. We can recommend this portion of the work to those of our readers who are engaged in the philosophy of the physiology of the blood, as an aid to investigation by presenting old facts in a new and altogether hypothetical form.

In the next section, Professor Schultz notices diseases of the organs of respiration, of the formative processes of animal life, or of the cerebro-spinal axis, and of soul-life in which latter are included morbid sleep, dreaming and somnambulism, and morbid spiritual assimilation and plastic.

In the eighth division, the external world as a cause of death is considered, and the author reviews in order the pathological relations of the atmosphere, and its elasticity, temperature, and electricity; the effects of light, and the action of climates, winds, &c. In perusing this portion of Professor Schultz's work we found ourselves breathing freely among more practical matter; and saw with pleasure, that the able Army and Navy Medical Reports, of which from time to time we have submitted a copious analysis to our readers, have been fully appreciated, and freely drawn upon by our author. We note one or two novelties in this section.

*How the atmosphere is purified.* It has been generally understood that plants purify the atmosphere by imbibing its carbonic acid and giving off oxygen. Professor Schultz has, however, instituted a series of researches into the physiology of plants (Die Entdeckung der wahre Pflanzen Nahrung nebst Aussicht zu einer Agricultur-physiologie, Berlin, 1844,) by which he shows that this is an error. According to his views, neither manures dissolved in carbonic acid, nor carbonic acid itself are assimilated by plants, but that humus is decomposed by the roots of plants into sugar and gum; that these are changed into vegetable acids, and hence the secretion of oxygen gas by vegetables takes place. It is quite true that in the light they give off the latter, but do not imbibe carbonic acid gas; on the contrary, the roots absorb oxygen gas, and in darkness the leaves, giving off, at the same time, carbonic acid and hydrogen gases. He shows also by these researches that the flowers of plants not only give off nitrogen, both by night and day, and absorb oxygen (a fact already established by Saussure,) but that they also secrete ammoniacal gas. So that in fact so far from rendering the atmosphere purer, plants render it impurer. Professor Schultz adds to these observations, that in winter, when vegetation is dormant, the air contains the least proportion of carbonic acid gas; when, according to the theory, it ought to contain the greatest; and that the latter makes no provision for the purification of the atmosphere from hydrogen, carburetted hydrogen, and sulphuretted hydrogen.

The true explanation is this. The extraneous gases are held in solution by the aqueous vapour contained in the atmosphere, and are precipitated with it in the form of rain. We can thus understand why the atmosphere over the sea is so free from carbonic acid gas; why in summer the feeling of freshness is perceived after a heavy rain; and also since the higher temperature of this season renders the aqueous vapour more copious, and the air more capable of holding this gas in solution, why there is more carbonic acid gas in the atmosphere in summer than in winter. Professor Schultz argues further that the compounds of hydrogen contained in the air are decomposed during thunder-storms by the lightning, the carburetted hydrogen being resolved into water and carbonic acid, the sulphuretted hydrogen into water and sulphuric acid. Heavy rains and thunder-storms are therefore the true purifiers of the atmosphere.

We had marked for notice some views of the author as to the laws of contagion, but we have only room for his general conclusion, that all diseases may under suitable circumstances become contagious. The proposition is supported by ingenious reasoning, but we think his arguments against the idea of a contagium animatum are altogether inconclusive.

We have now to state that this Second part is disfigured by some polemics aimed apparently at the author's famous competitor Schönlein—a novus homo in Berlin. We observe too that he quotes ourselves, implying that we had noticed his pathological views with approval, whereas we have not until now done more than place before our readers a brief account of his researches into the moults and renewals of the tissues, and accorded to those researches the merit of originality and profundity. The system of medicine which Professor Schultz has founded upon these and his other contributions to experimental philosophy is, we are certain, too hypothetical and too ideal, ever to be generally adopted by physicians. It will nevertheless give him rank amongst the medical philosophers of Germany, as in that country publications of this kind seem to be much more popular than in England or France.

---

